# Unusable cotton spinning mill waste: A viable source of raw material in paper making

**DOI:** 10.1016/j.heliyon.2022.e10055

**Published:** 2022-08-01

**Authors:** Sadikur Rahman, Ahmed Jalal Uddin

**Affiliations:** a Department of Textile Engineering, National Institute of Textile Engineering and Research, Nayarhat, Savar, Dhaka, Bangladesh; b Department of Yarn Engineering, Bangladesh University of Textiles, Tejgaon, Dhaka, Bangladesh

**Keywords:** Cotton waste, Filter waste, Hardwood kraft pulp, Handsheet, Writing and printing paper

## Abstract

Since the reserves of natural renewable resources are being inexorably diminished, the utilization of the recoverable waste in new area is gaining global attention day by day. Besides, as the cost of raw materials constitutes the majority of a production cost, the usage of undesirable but inevitable processing waste in the manufacturing process provides a considerable advantage to the manufacturers. Herein, it has been attempted to exploit unusable cotton spinning mill waste (filter waste derived from humidification plant) to convert it into paper. Handsheets of 70 g/m^2^ and 80 g/m^2^ were successfully produced from 100% cotton waste, 100% bleached cotton waste, and blends of bleached cotton waste with bleached hardwood kraft pulp (HWKP) (HWKP is typically used to produce commercial-grade papers). Morphologies and mechanical properties of handsheets were thoroughly investigated by whiteness index, brightness%, breaking length, tear index, bursting index, FTIR spectroscopy, optical microscope, and scanning electron microscope. Based on detailed observations, it is summarized that the produced handsheets, depending on the chemical treatment and blend ratio with HWKP, possess variations in appearances and properties that will have a wide range of potential applications from newsprint, tissue paper to commercial-grade writing and printing papers.

## Graphical abstract

**Figure:**
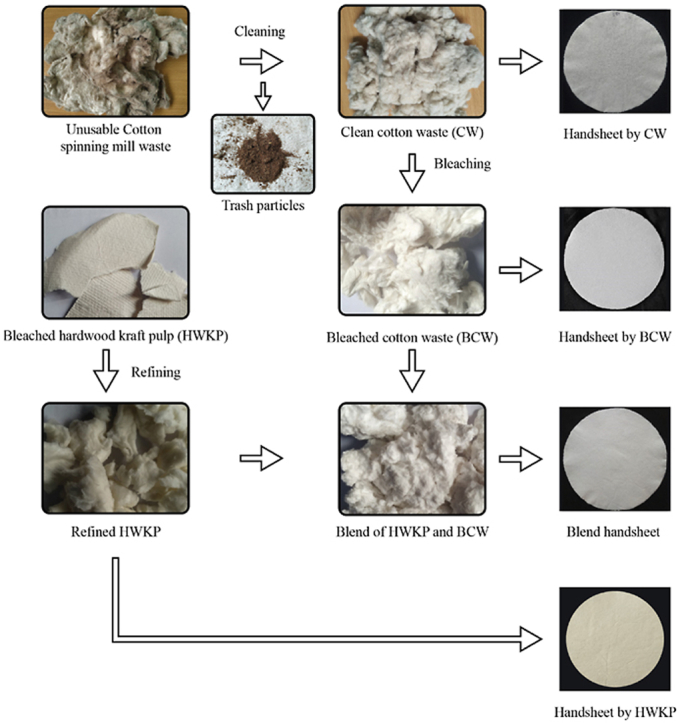

## 1 Introduction

Pulp and paper manufacturing has become an essential segment of world's industrial production. Paper plays an important role in the social, economic, and environmental development of any country. Paper manufacturing is typically based on the use of renewable natural raw materials like wood, non-wood, and recycled fibers (Bajpai, 2018). Rapid growth in population and industrialization have led to an increasing demand for high quality papers and paperboards (Abd El-Sayed et al., 2020) and their consumption may be projected at 461 million metric tons by 2030 (Tiseo, April, 2021). However, it is difficult to reduce the carbon and greenhouse gas emissions due to rapid surge in industrialization and urbanization. Consequently, strict legislations were imposed by the governments of developing and developed worlds to save environmental resources. For these reasons, pulp and paper industries have been facing a shortage of wood-based raw materials (Kaur et al., 2018). In this context, curbed production of forest-based materials along with the amplified demand for pulp and paper production, and environmental fortification awareness have enforced to find out alternate raw material (Ali and Sreekrishnan, 2000; Sharma and Kumar, 1999). Nowadays bagasse, rice straw, wheat straw, kenaf, jute, and hemp are considered as non-wood materials in making of paper (Kaur et al., 2017; Sridach, 2010) showing better pulp ability, good bleaching ability and excellent fiber content (Atchison, 1995; Jiménez et al., 2008).

**Figure 1:**
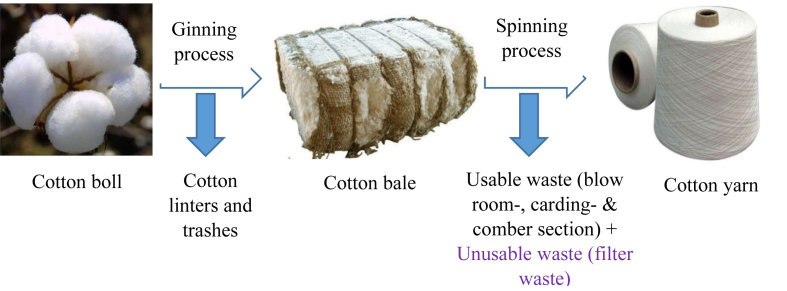
Cotton processing from fiber to yarn showing the removal of different wastes.

Cotton is a plant-derived natural seed fiber extensively used in the textile industry to manufacture yarns. The cotton linters are regarded as a valuable by-product of cotton processing and are used as an alternative source of cellulosic raw material in the pulp and paper industries (Sczostak, 2009). During the processing of cotton in spinning industries, around 8% wastes are generated in the blow room and carding section, and up to 20% noil in the combing section (Alagirusamy, 2013; Ute et al., 2019). These wastes are used in rotor spinning to manufacture coarser yarns for denim and jeans (Halimi et al., 2007). In addition, more than 1% cotton fly is produced by different machines throughout the processing in spinning line (shown in Figure 1) that is collected through the humidification plant, known as filter waste.

In 2019, the estimated total global fiber consumption was 108 million tons of which 25% share was of cotton fibers (Garside, 2020). Annually around 27 million tons of cotton fibers are produced all over the world (Dochia et al., 2012; Khan et al., 2020). As per 1% cotton fly generated during different processing in spinning industries, around 0.27 million tons of filter waste are produced worldwide per year. The collected filter waste is usually disposed of for landfill or directly burned into air that emits CO_2_ to the environment (Rajput et al., 2012). Bangladesh currently has 424 spinning mills and the sector is expanding fast in order to cope with the rising order of garments from USA, UK, Canada, Australia and many EU countries. In 2019, according to Bangladesh Textile Mills Association (BTMA), around 1.8 million tons of raw cotton were imported in Bangladesh for manufacturing yarns (BTMA, January 2020) and if 1% filter waste is assumed to be collected from these, then it turns to be total 18,000 tons filter waste per year. If this quantity of filter waste can be used as a raw material or blend constituent in pulp and paper industry, this will be equivalent to the reduction of imported pulp used during bulk production. For example, using 20% spinning filter waste in paper products will essentially reduce 20% imported pulp consumption during its production.

The current work was undertaken to utilize, for the first time, the unusable filter waste of the spinning mill as a raw material for paper manufacturing. Handsheets were prepared from 100% cotton waste, 100% bleached cotton waste, and blends of bleached cotton waste with bleached hardwood kraft pulp (HWKP). The morphological and mechanical properties of the prepared handsheets were compared with a reference handsheet manufactured with 100% HWKP that is traditionally used to produce commercial-grade writing and printing papers.

## 2 Materials and methods

### 2.1 Materials

**Figure 2:**
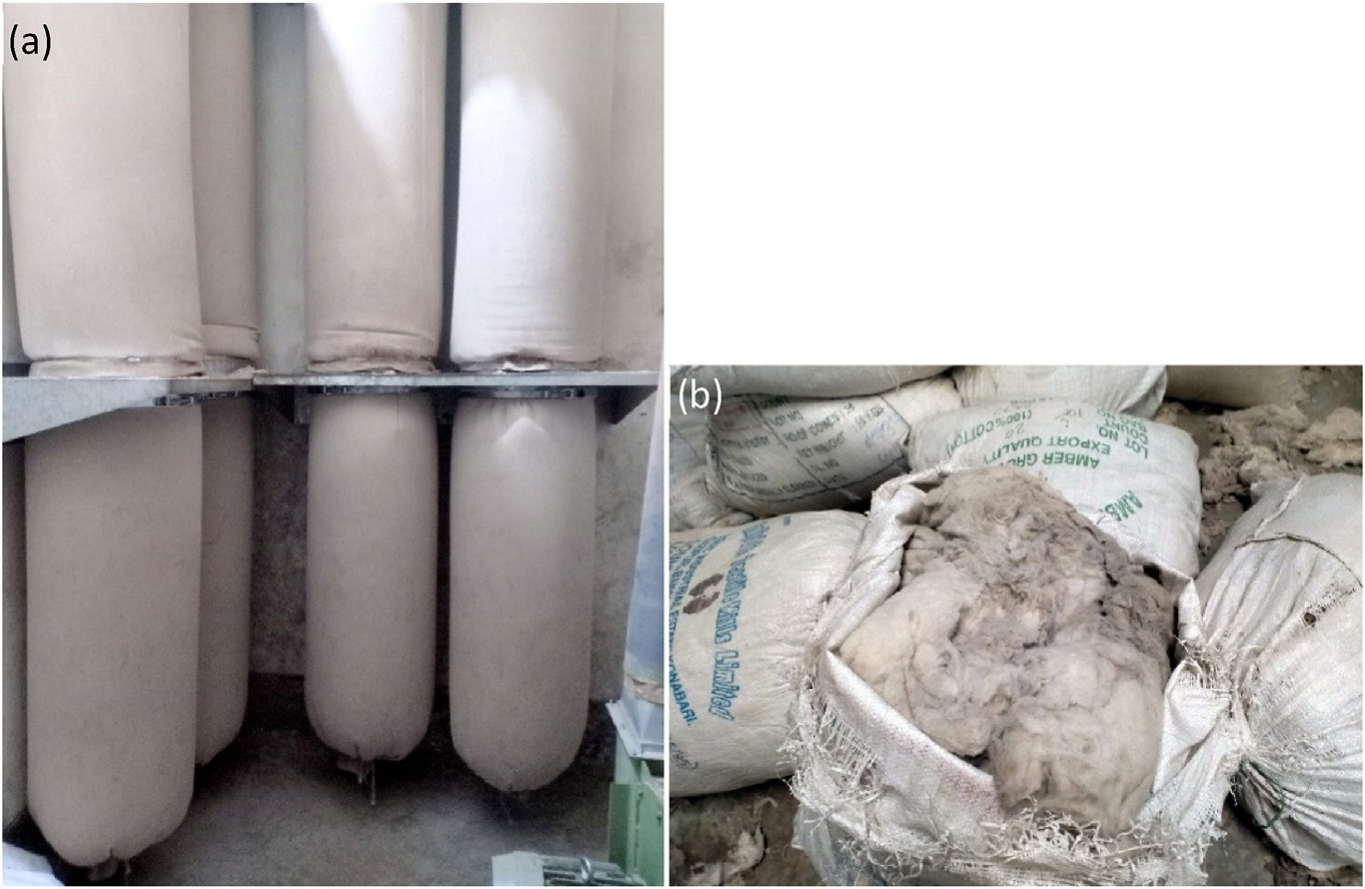
(a) Filter waste collector of humidification plant of the spinning mill, and (b) filter waste obtained from it.

Unusable cotton waste of spinning mill, commonly known as filter waste, accumulated in filters of humidification plant (shown in Figure 2) was collected from Maksons Spinning Mills Ltd, Dhaka, Bangladesh. The collected waste was then passed through a Shirley Analyzer to remove the impurities or non-fiber substances. In order to prepare a reference handsheet made of bleached hardwood kraft pulp (HWKP), HWKP, imported from Indonesia through M/S Virtuas Sourcing Bangladesh, was procured from Partex Paper Mills Ltd, Narayanganj, Bangladesh. Length, breaking length, diameter and brightness of refined fibres were below 2 mm, 5000–7000 m, 10.7 μm and 83.59%, respectively.

#### 2.1.1 Chemicals

Lab grade chemicals of Sodium hydroxide (NaOH), hydrogen peroxide (H_2_O_2_), acetic acid (CH_3_COOH), and sodium thiosulfate (Na_2_S_2_O_3_) were collected from Kuri & Company (Pvt.) Ltd. which was the authorized distributor of Sigma Aldrich in Bangladesh.

### 2.2 Preparation of handsheets

**Figure 3:**
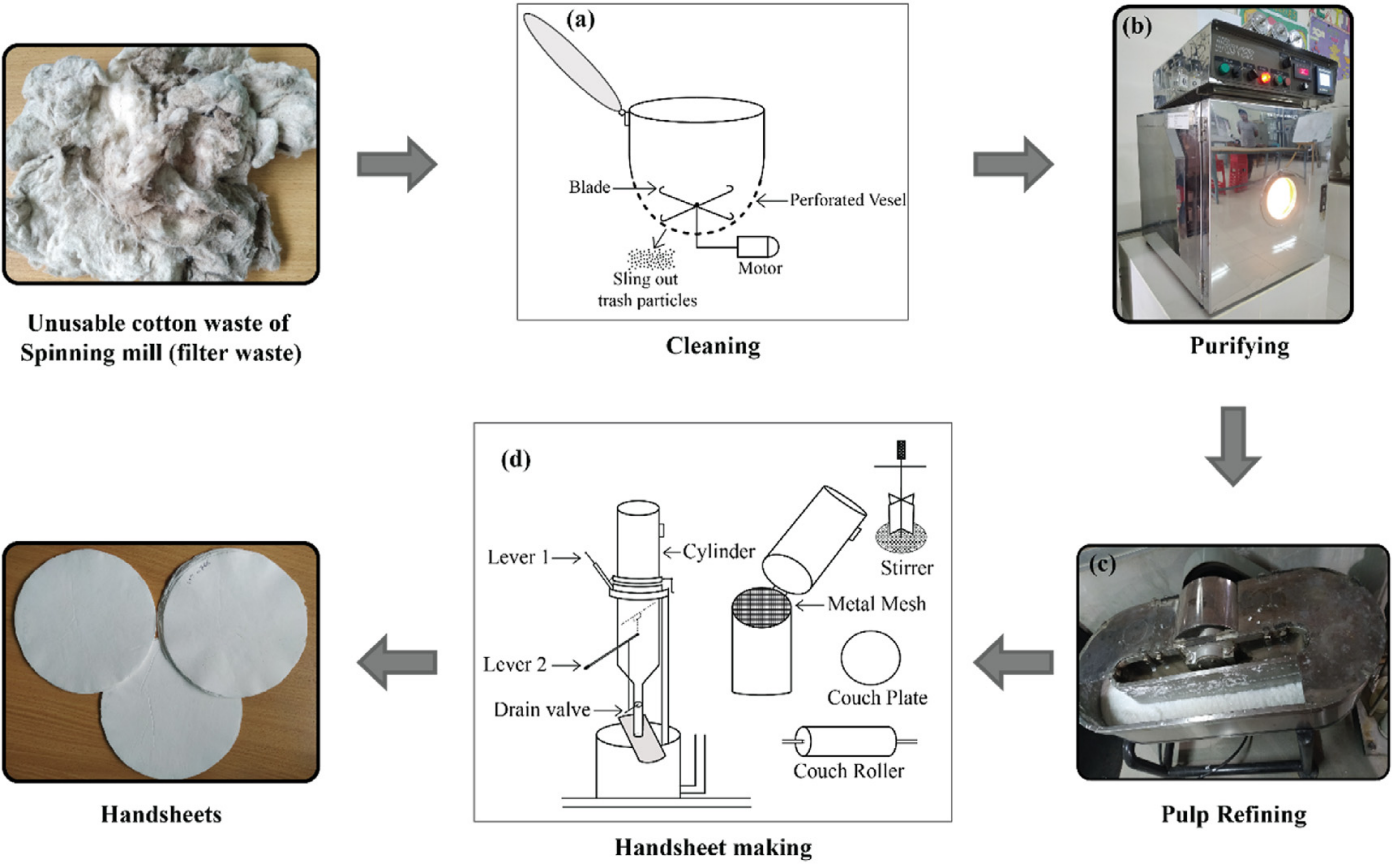
Differen**t** steps of handsheet preparation.

According to TAPPI Standard T 205, a handsheet is a single sheet of circular paper with 15.9 cm (6.25 in) in diameter prepared by a hand process. The purpose of handsheet preparation is to determine the quality parameters of paper to be made from a given batch of pulp in a short time. Different steps to prepare the handhseet from filter waste are illustrated in Figure 3.

#### 2.2.1 Cleaning of cotton waste

The prime objective of the cleaning process was to separate the impurities like the fine seed, leaf particles and contaminations like colored fibers, bird feathers, and polyethylene from cotton waste. After manual separation of contaminations, a specially designed cleaning machine was used to remove the seed particles as shown in Figure 3a. The machine consists of two blades with a perforated body which permits the seed particles to sling out. Motor capacity was 1,000 Watt having a controlling beater speed from 12,000–18,000 rpm. After cleaning the cotton waste, fiber yield was found to be around 75%. The fiber length was measured by Shirley Comb Sorter and observed 2–6 mm. The fiber diameter was ascertained from optical microscopic image using ImageJ software and it was 12.1μm on average. Fiber strength was measured by Stelometer with zero gauge length and found 3–5 cN/tex.

#### 2.2.2 Purification of cotton waste

**Figure 4:**
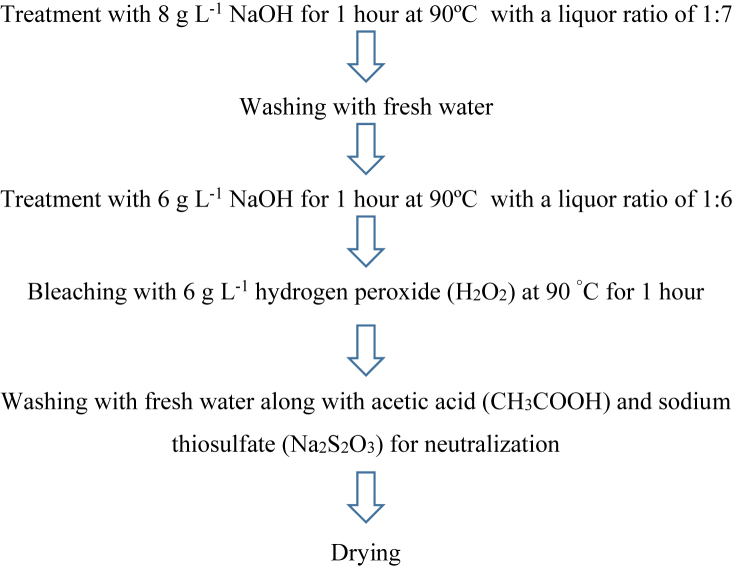
Different stages of purification of cotton waste by chemical treatment.

The aim of the purification of cotton waste by different chemicals was to remove its different impurities like protein (1.0–2.1%), wax (0.4–1.7%), ash (inorganic salts) (0.7–1.8%), pectin (0.4–1.9%), and others (resins, pigments, hemicelluloses) (1.5–2.5%) ranging from 4% to 10% (Gallo and Almirall, 2009; Plant et al., 1998). The amount of impurities in raw cotton fiber depends on its type, origin, fiber maturity, weathering, and agricultural conditions (Mandal et al., 2005; Stathakos et al., 2006). Cotton fibers are usually treated with sodium hydroxide (NaOH) to remove natural oil, wax, protein and pigments that improves the wettability and dressing performance (Bulut, 2016; Ghule et al., 2004; Wang et al., 2020). However, purification process in the current work was carried out in a Mathis Infra-red dyeing machine (shown in Figure 3b) that is exhibited by the block diagram in Figure 4 (Mustajoki et al., 2010).

After purification by chemical treatment, yield of clean fiber was around 88% of clean cotton waste. The fiber length, diameter and strength were measured to be 2–3 mm, 13.7 μm and 2–3 cN/tex, respectively.

#### 2.2.3 Cutting, soaping, and drying

Bleached cotton waste was subjected to cut manually into the long fibers and beaten in a vessel with hot water (90 °C) for fibrillation. Then the fibers were crumbed and soaked with water easily (Gharehkhani et al., 2015). Then water was drained out and the fibers were collected from the vessel as a bundle and dried in sunlight.

#### 2.2.4 Pulp refining

As shown in Figure 3c, the fiber shortening, straightening, fine formation, internal fibrillation, and swelling were carried out through the valley beater (Garcia et al., 2002; Jones et al., 2013; Park et al., 2006). At first, the valley beater was run with 360 g dry cotton waste with 23 L water for 25 min without adding weight which caused only water flow inside the valley beater to mix the cotton waste or soak with water. Secondly, the machine was run by adding weight (6 kg) which caused compressive beating action to achieve the degree of refining. The degree of refining was found through the Schopper-Riegler (SR) test which indicates the speed of the drainage of the diluted cotton suspension according to ISO 5267–1:1999. Cotton waste was refined at different levels and measured SR degree for which the maximum breaking length of the handsheet was achieved. Here, 35^°^ SR was obtained by operating valley beater for 30 min which provides maximum breaking length. After refining, fiber length and diameter were measured from SEM images and found to be 0.07–0.29 mm and 3.9–13.2 μm, respectively. Imported HWKP was also refined through the valley beater. Machine was run for around 2.5 h to achieve 30^°^ SR.

#### 2.2.5 Feedstock preparation

Cotton waste, bleached cotton waste, and bleached hardwood kraft pulp were used to produce 5 kinds of handsheets as shown in Table 1.

**Table 1:** Name and percentage of feed material to produce 5 kinds of handsheets

Type	Feed material	Percentage
1.	Bleached hardwood kraft pulp (HWKP)	100%
2.	Cotton waste (CW)	100%
3.	Bleached cotton waste (BCW)	100%
4.	Blend of bleached hardwood kraft pulp with bleached cotton waste (50/50 blend of HWKP/BCW)	50/50%
5.	Blend of bleached hardwood kraft pulp with bleached cotton waste (75/25 blend of HWKP/BCW)	70/25%

#### 2.2.6 Preparation of handsheet

As schematically shown in Figure 3d, a handsheet former machine (Pap Tech Engineers & Associates, Jaipur, India) was used to make handsheets according to laboratory handsheets making method ISO 5269–1:2005 where pulp concentration was 2.47%. A solution was made by refined soaked pulp with water by adding 1% (dry weight of pulp) alkyl ketene dimer (AKD) which assisted the sizing of the handsheet and then stirred by the stirrer. After making handsheets, a press was used to apply an even pressure of 400 ± 10 kPa for 5min ±15 s. Then the handsheet was dried in air at lab temperature for 12 h. After drying, the separation of handsheet from the blotting paper was carried out very carefully. By this process, 5 kinds of handsheets of 70 g/m^2^ and 80 g/m^2^ each were produced. The demands for 70 and 80 g/m^2^ writing and printing papers are high in the market that is usually produced with the HWKP pulp. In order to compare the properties, imported HWKP pulp was used here to produce a reference handsheet and thus 70 and 80 g/m^2^ handsheets were manufactured for all samples.

##### 2.2.6.1 Scanning electron microscopy (SEM) and optical microscopy

The surface morphology of the handsheets was examined using scanning electron microscope (SEM), FEI Inspect S-50, Japan with accelerating voltage 15 kV. From SEM images, the average fiber length and diameter were ascertained by using ImageJ software.

The appearance of fibers at the torn edge of the handsheet with paraffin was observed by an optical microscope (Leica DM4 P, Germany).

### 2.3 Physical characterization of paper sheets

In order to study the mechanical properties of handsheets, all the samples were first pre-conditioned and then conditioned according to TAPPI T402 as the moisture content has a significant effect on paper strength and other properties (Biermann, 1996).

#### 2.3.1 Optical properties

Paper whiteness indicates its ability to equally reflect a balance of all light wavelengths across the visible spectrum (approximately 380 nm–720 nm). Handsheet appears to be white if it reflects light equally at all wavelengths across the visible spectrum. If some wavelengths are absorbed while others are reflected, the object exhibits the color of the reflected light color that can be measured. Therefore, pure white is an achromatic reflector of perfectly balanced light (Bristow, 1994; Norberg, 2007). The whiteness of a handsheet was measured by Datacolor ms D650 according to ISO 11475:2004 which specifies a whiteness index. Its measurement method uses diffuse illumination with a light source configured to CIE standard illuminant D65. CIE whiteness is a single number value expressed as whiteness units or whiteness index (WI) (0–100). But whiteness index may be above 100 if fluorescent components are used for high-quality papers (ISO, 2004). The brightness is the percentage of blue light reflected from the surface of paper at a specific wavelength of 457 nm (full width at half maxima is 44 nm). The brightness of paper is measured on a scale of 0–100% – the higher the number, the brighter the paper. Here, the brightness percentage of handsheet was measured according to TAPPI Test Method T452 (Ragauskas, 2020).

#### 2.3.2 Mechanical properties

After conditioning the handsheets, strength and other physical tests were carried out according to TAPPI T 205 “Forming Handsheets for Physical Tests of Pulp”. Here the tensile tests were conducted by using a universal strength tester (FRANK-PTI GmbH, Germany) with a 500 N load cell at 50 mm/min speed. The breaking length is the length of a strip of paper required to break the strip under its weight. It can be calculated using the following relationship:

$$\text{Breaking}\;\text{Length}\;\left(\text{km}\right)=102\times \frac{\text{Tensile ​Strength ​}\left(\text{kN}/\text{m}\right)}{\text{Grammage ​}\left(\text{g}/m^{2}\right)}$$

Tearing resistance was ascertained by an Elmendorf resistance type tear tester (Pap Tech Engineers & Associates, Jaipur, India) according to TAPPI T 414 om-98. Bursting strength was measured by a Mullen type bursting strength tester (Pap Tech Engineers & Associates, Jaipur, India) according to TAPPI T 403 om-97. All experimental results were evaluated as averages of 10 measurements.

#### 2.3.3 FTIR spectroscopy

Infrared spectra of handsheets were recorded with a PerkinElmer FT-IR, UATR Two (Model: SL No. 109502), UK. The influence of CO_2_, moisture, and oxygen in air was eliminated by measuring the background spectra before each measurement. Bands were recorded in the region from 4,000-400 cm^−1^.

## 3 Results and discussion

### 3.1 Visual appearance of handsheets

**Figure 5:**
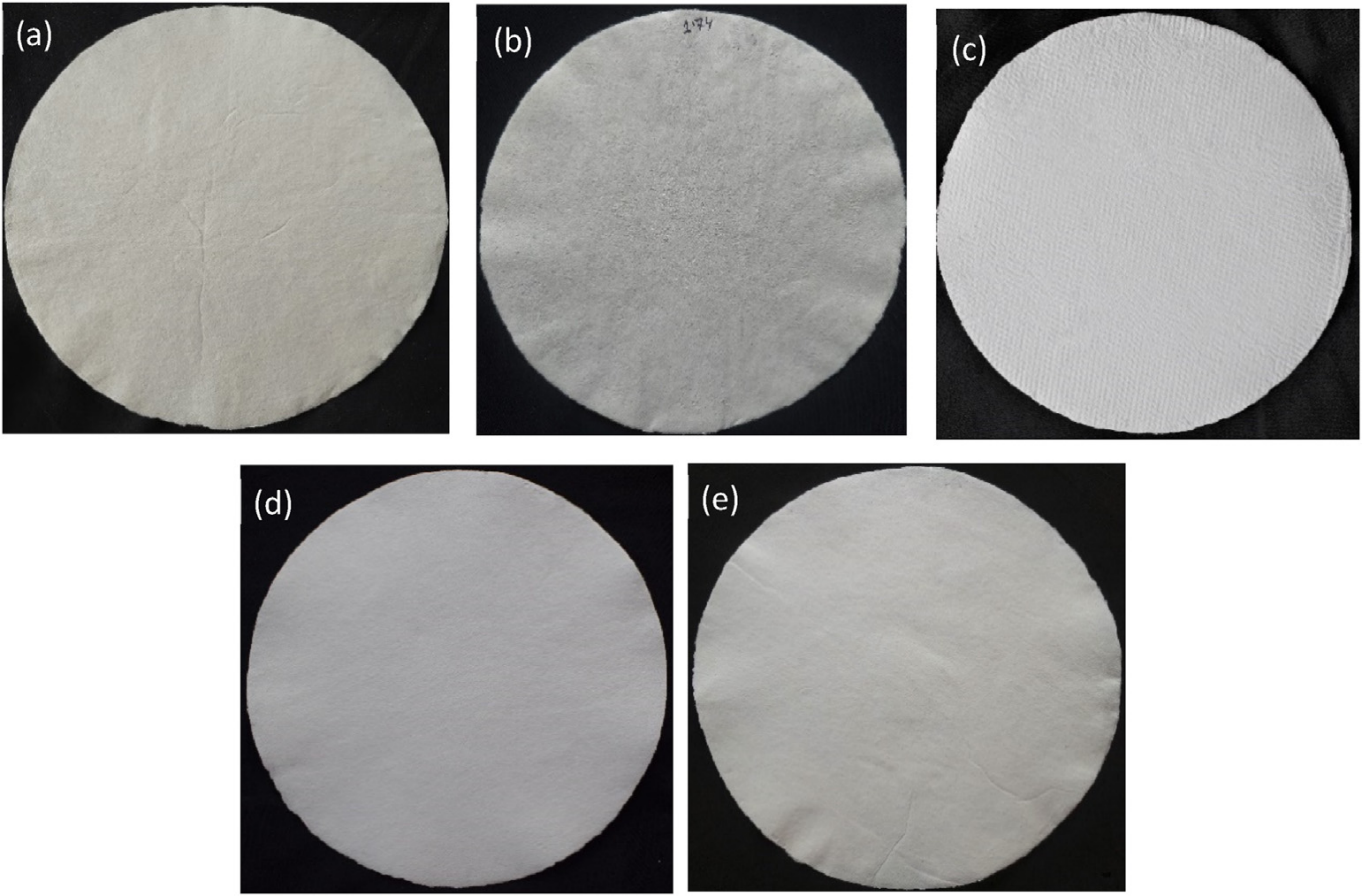
Images of handsheets made with (a) bleached hardwood kraft pulp (HWKP), (b) cotton waste (CW), (c) bleached cotton waste (BCW), (d) a 50/50 blend of HWKP/BCW, and (e) a 75/25 blend of HWKP/BCW.

Images of 5 kinds of handsheets produced with bleached hardwood kraft pulp (HWKP), cotton waste (CW), bleached cotton waste (BCW), a 50/50 blend of HWKP/BCW, and a 75/25 blend of HWKP/BCW are shown in Figure 5.

As mentioned in experimental section, a handsheet from bleached HWKP was produced to use as a reference material to compare its properties with the produced handsheets in the current work. As seen in Figure 5a, handsheet prepared from bleached HWKP looks slightly yellowish. In Figure 5b, handsheet prepared by CW is darker than other handsheets as it was not bleached. CW was subjected to bleaching to achieve whiteness to produce writing and printing grade paper as done by HWKP. Resultantly, handsheet prepared by BCW, shown in Figure 5c, is quite whiter. Whiteness of handsheets prepared from 50/50 blend of HWKP/BCW (Figure 5d) and 75/25 blend of HWKP/BCW (Figure 5e) decreases according to the decreasing proportion of BCW.

### 3.2 Whiteness index and brightness% of handsheets

**Figure 6:**
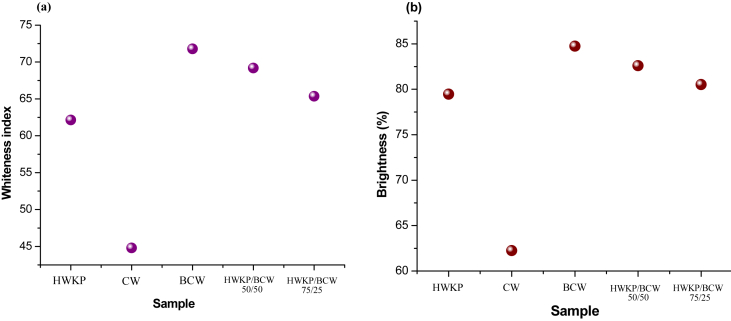
(a) Whiteness index and (b) brightness of handsheets prepared with bleached hardwood kraft pulp (HWKP), cotton waste (CW), bleached cotton waste (BCW), 50/50 blend of HWKP/BCW, and 75/25 blend of HWKP/BCW.

The whiteness index and brightness% of different handsheets are shown in Figure 6a and Figure 6b. The whiteness index and brightness of reference handsheet prepared from HWKP were found to be 64 and 79.5%. Compared with the reference handsheet, markedly lower whiteness index (44.80) and brightness (62.28%) were found for the handsheet prepared from cotton waste (CW). This handsheet was comparatively darker and yellowish as it was unbleached and contains fine trashes (Gallo and Almirall, 2009; Plant et al., 1998) and protoplasmic residues of protein and the flavones pigments of cotton (Abidi et al., 2010; Ghule et al., 2004). Handsheet made of bleached cotton waste (BCW) was whiter and bluer that shows substantial increase in whiteness index (71.80) and brightness (84.75%) resulted from scouring and bleaching of cotton waste (Abdel-Halim, 2012). This kind of bleached and total chlorine free (TCF) non-wood pulp has an eminent application effect and prospect in paper manufacturing (Liu et al., 2018).

The whiteness index and brightness of blend handsheets i.e. 50/50 HWKP/BCW and 75/25 HWKP/BCW showed lowering trend in accordance with the decrease of the ratio of BCW, but still higher than those of the handsheets prepared individually from HWCP and CW. Therefore, BCW can be used as a blend constituent with HWKP to control the whiteness index and brightness to produce the required grade of writing and printing paper.

**Figure 7:**
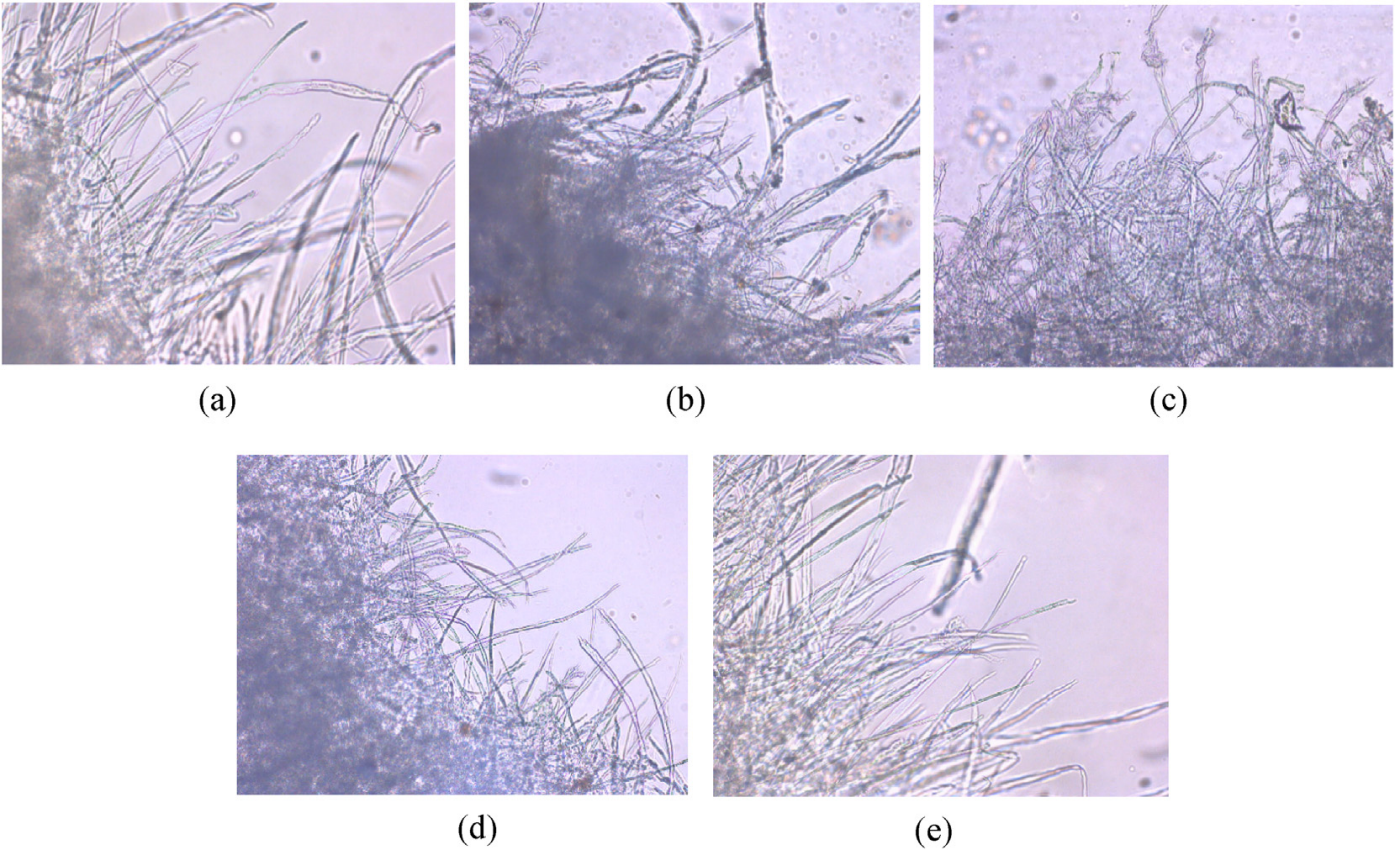
Optical microscopic images (20×) of handsheets prepared from (a) 100% bleached hardwood kraft pulp (HWKP), (b) cotton waste (CW), (c) bleached cotton waste (BCW), (d) 50/50 blend of HWKP/BCW, and (e) 75/25 blend of HWKP/BCW.

The appearance of fibers at the torn edge of the handsheets observed by an optical microscope is shown in Figure 7. It is seen that the handsheets made of cotton waste (CW) and bleached cotton waste (BCW) have a low number of long fibers at the torn edge (Figures 7b and 7c, respectively). The handsheet of CW (Figure 7b) appears darker and has many dark spots on the fiber surface. But the sample of BCW (Figure 7c) is brighter with no dark spots on the fiber surface due to chemical treatment. Many illuminated long fibers with no dark spot on the fiber surface appear at the torn edge in the handsheet made with hardwood kraft pulp (HWKP) (Figure 7a) as the wood pulp was chemically purified and chipped into the desired length. Compared with the handsheet made of 100% HWKP (Figure 7a), the 50/50 blend of HWKP/BCW handsheet (Figure 7d) appears brighter and shows a mixture of long and short fibers as the handsheet contains 50% HWKP and 50% BCW. Handsheet prepared with 75/25 blend of HWKP/BCW (Figure 7e) exhibits more illuminated long fibers as it contains 75% HWKP.

### 3.3 Thickness and mechanical properties of handsheets

**Figure 8:**
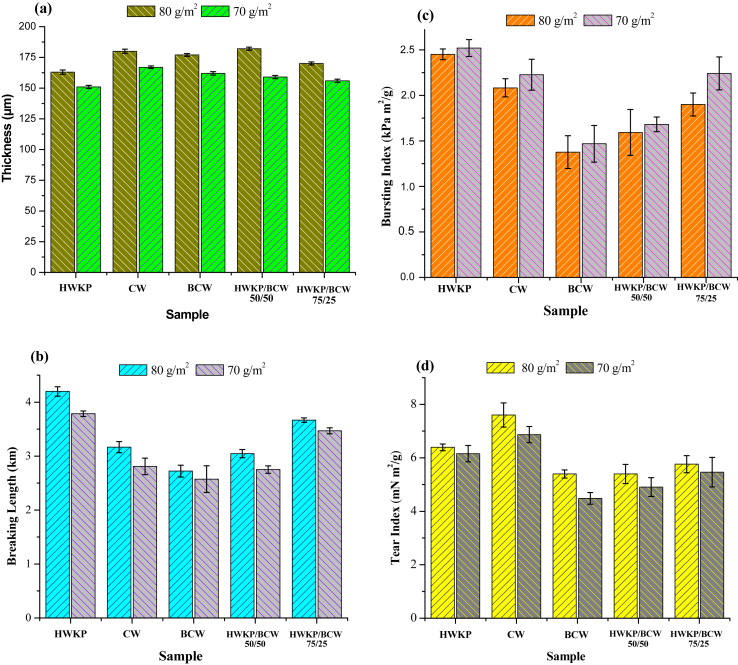
(a) Thickness (b) Breaking length, (c) Bursting index, and (d) Tear index of handsheets produced with bleached hardwood kraft pulp (HWKP), cotton waste (CW), bleached cotton waste (BCW), 50/50 blend of HWKP/BCW, and 75/25 blend of HWKP/BCW.

The thickness of the handsheets is shown in Figure 8a. It can be seen that the thickness of the handsheet of CW and BCW for 70 g/m^2^ and 80 g/m^2^ was slightly higher than the handsheet of HWKP due to swelling of the cotton fiber. The blend of 75/25 HWKP/BCW showed a comparable thickness with the same of handsheet produced with bleached HWKP.

The mechanical properties of handsheets are presented in Figures 8b – 8d. The handsheet produced from bleached hardwood kraft pulp (HWKP) shows the highest breaking length and bursting index. The handsheet of bleached cotton waste shows the lowest breaking length, bursting index, and tear index resulted from the generation short fibres and fines during chemical treatment and beating action to obtain 35° SR in order to achieve maximum fiber properties (Nazhad et al., 2000; Saikia et al., 1997). Fines are very undesirable elements in stock during paper making since their presence reduces freeness and increases water retention of pulp. The presence of fines in large numbers hampers the inter-fiber bonding in paper (Kamoga et al., 2016). The individual fiber characteristics such as length, strength, structure, and arrangement of fiber bonding to each other affect the breaking length of handsheets (Gonzalo et al., 2017) and handsheets prepared from long fibers show higher mechanical properties than those from short fibers (Fagbemi et al., 2017; Tutus et al., 2010) as shown in Figure 8. In Figure 8d, handsheet from cotton waste showed higher tear index than others handsheets because fibrillation of cotton waste was carried out without any chemical treatment. In the case of hardwood pulp fibers, hardwood was refined for pulp making. Hence the amount of smaller fiber size and decrease in the fiber length were lower than those of pulp produced from cotton waste leading to slightly smaller tear index. Handsheets prepared from bleached cotton waste, hardwood kraft pulp and their blends also showed lower tear index as they were subjected to chemical treatment for purification.

As shown in Figure 8c, the handsheet from cotton waste presents a better bursting index than the handsheet of BCW and blends of HWKP/BCW. In the case of blend handsheets, an increase in the amount of BCW with HWKP leads to decrease in breaking length, tear index, and bursting index. The 75/25 HWKP/BCW handsheet shows a better mechanical properties than 50/50 HWKP/BCW handsheet and nearly to the result of handsheet made by HWKP.

**Figure 9:**
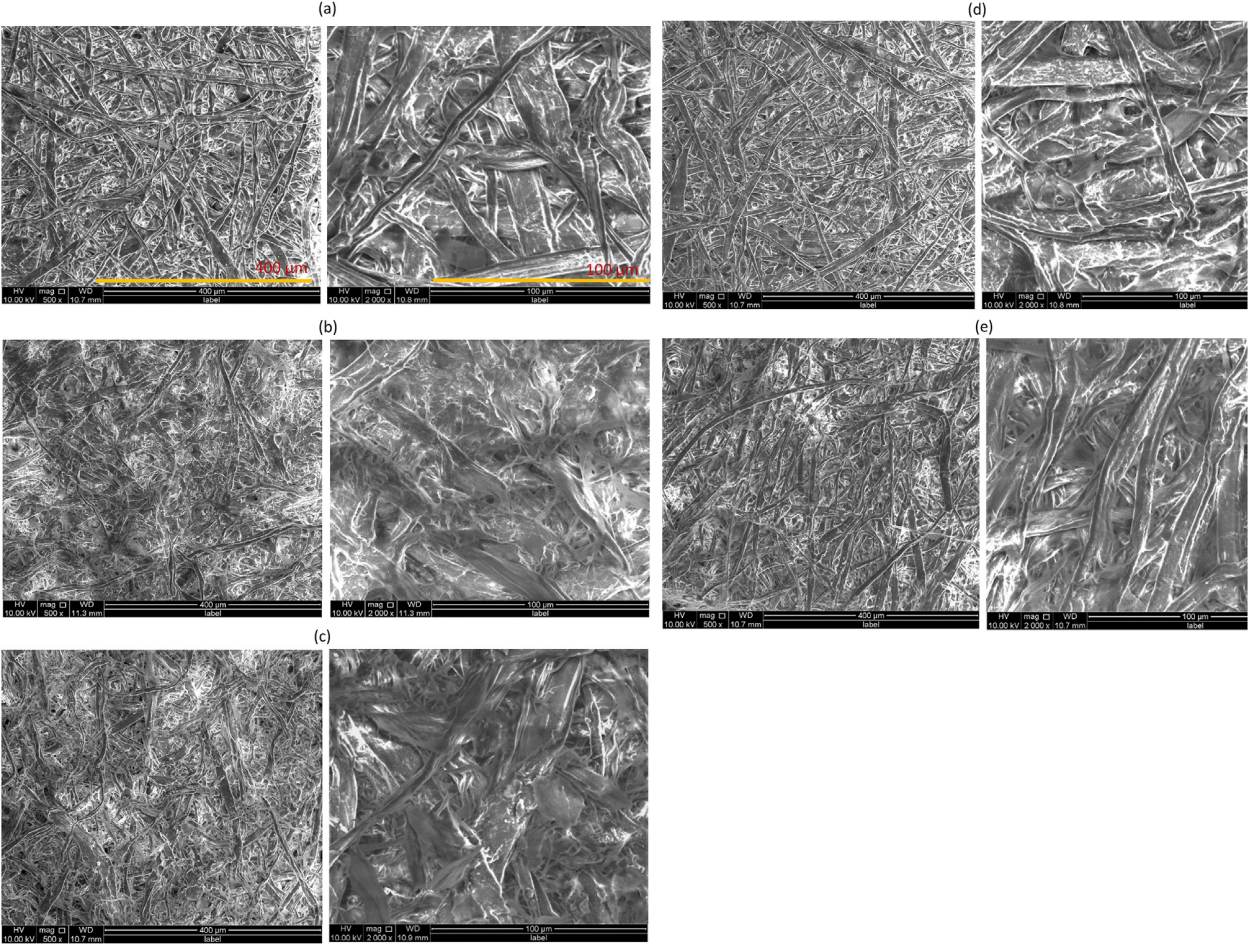
SEM images of handsheets prepared with (a) 100% bleached hardwood kraft pulp (HWKP), (b) cotton waste (CW), (c) bleached cotton waste (BCW), (d) 50/50 blend of HWKP/BCW, and (e) 75/25 blend of HWKP/BCW magnified at 500× (left) and 2000× (right).

SEM images of all handsheets are shown in Figure 9. It is seen that the surface of the handsheets made by CW (Figure 9b) and BCW (Figure 9c) appears to have smooth surfaces with linking like gel-like material. They have a low number of long fibers with lengths ranging 220-70 μm and diameters ranging 13.2–3.9 μm with less porous surface morphology. Maximum fibers on the surface of both handsheets were collapsed with increased contact area and fibrillation that made the sheet network more compact. The surface of the handsheet made by HWKP (Figure 9a) is rough and has many tubular shape long fiber structures having lengths 502-217 μm that are randomly organized. The sheet network has plenty of empty spaces and the diameters of fibers were between 21 μm to 6 μm. But both blend handsheets made of HWCP/BCW (Figures 9d and 9e) show the coherence of fibers with less cavity among the long fibers. Here the BCW, in both blend handsheets, acts as a filler that enhanced the interaction of fibers by filling the empty spaces between fibers which helps to create better fibrillation, contact area, smooth surface, less porous structure than the handsheet made of 100% HWKP.

On the basis of the mechanical properties, whiteness index and brightness% (Figures 6a and 6b), cotton waste may be regarded as a new raw material to manufacture newsprint grade paper and packaging material. Bleached cotton waste may be used to produce specialty papers like security paper, document paper and filter paper as done with cotton linters by (Abd El-Sayed et al., 2020). By adjusting the ratio of HWKP and BCW, blend handsheets may be utilized to produce a wide range of writing and printing papers.

### 3.4 FTIR spectroscopy

**Figure 10:**
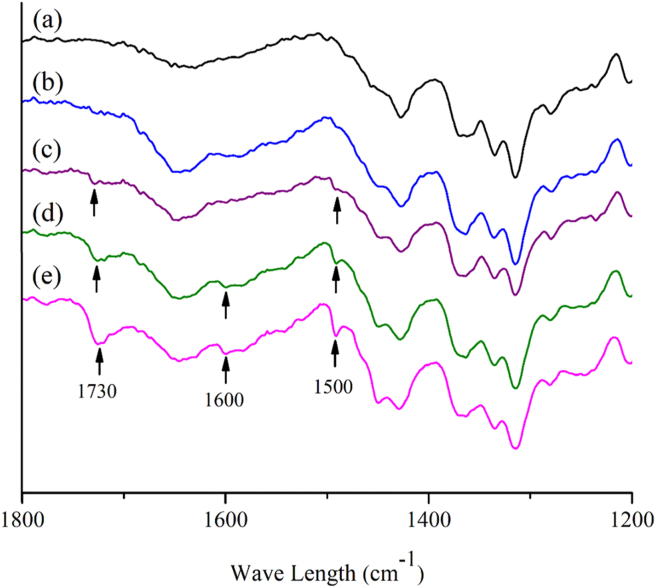
FTIR spectra for (a) Cotton waste (CW), (b) Bleached cotton waste (BCW), (c) 50/50 blend of HWKP/BCW, (d) 75/25 blend of HWKP/BCW and (e) 100% bleached hardwood kraft pulp (HWKP).

FTIR spectra of handsheets are shown in Figure 10. In the case of handsheet sample of 100% bleached hardwood kraft pulp (HWKP) (Figure 10e), the bands appeared at approximately 1730, 1600 and 1500 cm^−1^ correspond, respectively, to the ester linkage of the carboxylic group of the ferulic acid and p-Coumaric acids of lignin, C=C aromatic ring of lignin, and aromatic ring of lignin (Adel et al., 2016; Uddin et al., 2010). In the case of blend handsheets such as 75/25 blend of HWKP/BCW (Figure 10d) and 50/50 blend of HWKP/BCW (Figure 10c), the magnitudes of lignin bands decrease with the decrease of HWKP content in blend handsheets. Since there is no lignin in cotton, no equivalent bands are found for the samples made with cotton waste (CW) and bleached cotton waste (BCW) (Figures 10a and 10c) (Wakelyn et al., 2006). The advantages of making handsheets from lignin-free fibres i.e. cotton in the current work are mentioned below.

### 3.5 Cost considerations for paper production

The spinning industry generates a substantial amount of unusable cotton filter waste and in Bangladesh alone, its amount is as much as 18,000 tons per year (mentioned in Introduction section). Compared to pulp making from softwood and hardwood, pulping from non-wood plants requires one-third of time, 30% less chemical, and reduced power due to lower lignin content (Haile et al., 2021; Kazi, 2018; Ververis et al., 2004; Young, 1997). More importantly, cotton fibres are lignin-free (as shown in Figure 10a) and can easily be converted into pulp. Bleaching of cotton fiber is also easier (Topalovic et al., 2007). However, about 60,000–1,00,000 gallons of water are required for the production of one ton of paper from hard wood that releases more than 47,000–80,000 gallons of waste water consisting of lignin and bleaching agents like chlorophenols (Dwivedi et al., 2010; Dixit et al., 2020). But in the current study (mentioned in section 2.2.2), the chemical treatment to convert CW to BCW was carried out by using only 11000 gallons water with 106 kg NaOH and 42 kg hydrogen peroxide (H_2_O_2_). The above information substantiates that the pulp production from cotton waste significantly saves the production cost as well as the environmental pollution.

Bangladesh has over 100 paper mills with a capacity of producing over 1.5 million tons of paper and paper products annually (Parvez, 2020). The major drawback in the developing paper industry in Bangladesh is the need for importing raw materials (Jahan et al., 2009). In Bangladesh, paper mills directly import wood pulp as a raw material for manufacturing paper. In 2018, the prices of imported hardwood pulp was reportedly increased to $810 from $470/ton and to $950 from $500/ton for softwood pulps (Uddin, 2018). In the context of current work, approximately $300/ton will be the cost for pulp manufacturing as shown in Table 2 from cotton waste in Bangladesh. The above information means, in general, a significant lower cost of paper pulp production from unusable spinning cotton waste. In Bangladesh, around 30–35% of production costs can be minimized if the cotton waste pulp is used as a blend constituent with hardwood kraft pulps for paper manufacturing.

**Table 2:** Pulp manufacturing cost from unusable cotton spinning mill waste.

Sl. no	Particulars	Cost per ton	Particulars	Cost per tones
	**A. Direct Cost (material)**		**B. Indirect Cost**	
1	Raw material (filter waste) price	$ 20	Transport∗	$ 30
2	Material processing cost	$ 100	Utility	$ 20
3	Purifying chemicals	$ 75	Maintenance	$ 10
4	Soaping and drying cost	$ 35	Storing	$ 10

Total Cost, C = (A + B) = $ 335.

∗ Transportation costs may vary from place to place.

## 4 Conclusions

The current work showcases a pathway to valorize unusable cotton spinning mill waste in paper products. Handsheets from 100% unusable cotton spinning waste (CW), 100% bleached cotton waste (BCW), and a blend of bleached hardwood kraft pulp (HWKP) and bleached cotton waste (BCW) were successfully produced. Compared with the commercial-grade handsheet made with HWKP, the handsheet produced from 100% CW showed a remarkably lower whiteness index and brightness% whereas it was noticeably higher in the case of 100% BCW. Handsheets obtained from blends of HWKP/BCW showed an intermediate whiteness index and brightness% depending on the ratio of BCW. Concerning mechanical properties, 100% CW handsheet showed slightly lower breaking length and bursting index but higher tear strength. 100% BCW handsheet exhibited the lowest breaking length, bursting index, and tear index. Blend handsheets showed intermediate mechanical properties depending on the ratio of BCW. With regard to blend handsheet, it can be said that BCW can be used as a component to suitably blend with HWKP in order to control the brightness% and mechanical properties as well as the cost of handsheets.

The handsheets prepared from 100% unusable cotton spinning waste can be used to produce papers for newsprint, magazine and packaging. Handsheets prepared from 100% bleached cotton waste are suitable to produce hand towel tissue and toilet paper where strength is not a remarkable factor. Blend handsheets prepared from HWKP and BCW are suitable for commercial-grade writing and printing papers.

## Declarations

### Author contribution statement

Sadikur Rahman: Conceived and designed the experiments; Performed the experiments; Contributed reagents, materials, analysis tools or data; Wrote the paper.

Ahmed Jalal Uddin: Conceived and designed the experiments; Analyzed and interpreted the data; Contributed reagents, materials, analysis tools or data; Wrote the paper.

### Funding statement

This research did not receive any specific grant from funding agencies in the public, commercial, or not-for-profit sectors.

### Data availability statement

Data will be made available on request.

### Declaration of interest's statement

The authors declare no conflict of interest.

### Additional information

No additional information is available for this paper.
